# The Current Landscape of Clinical Trials

**DOI:** 10.3390/jcm14072519

**Published:** 2025-04-07

**Authors:** Geeta Joshi, Tara K. Bhandari, Pushkar Joshi, Smriti Bhandari, Shalini Reddy Araveeti, Aditi Jain, Subash Khadka, Shaun Trecarten, Ahmad Abdelaziz, Harshit Garg, Mukund Bhandari

**Affiliations:** 1 Department of Medical Education, UTHealth San Antonio, San Antonio, TX 78229, USA; 2 Department of Education, Kathmandu University, Dhulikhel 45200, Nepal; bhandaritara45@gmail.com; 3 Department of Management, Webster University, San Antonio, TX 78213, USA; pushkarjoshi@webster.edu; 4 School of Management, Tribhuwan University, Kritipur 44618, Nepal; 5 New England College, Henniker, NH 03242, USA; 6 Department of Reproductive Medicine, Sri Gangaram Hospital, Delhi 110060, India; aditijain1205@gmail.com; 7 Department of Cell Systems & Anatomy, UTHealth San Antonio, San Antonio, TX 78229, USA; khadka@uthscsa.edu; 8 Department of Urology, UTHealth San Antonio, San Antonio, TX 78229, USA; trecarten@uthscsa.edu; 9 St. Elizabeth Medical Center, Boston, MA 02135, USA; ahmedmfaziz92@gmail.com; 10 Department of Urology Oncology and Robotic Surgery, Max Institute of Cancer Care, Delhi 110024, India; hgarg3108@gmail.com; 11 Department of Pathology, UT Southwestern Medical Center, Dallas, TX 75235, USA

**Keywords:** clinical trials, randomized controlled trials, clinicaltrials.gov, clinical trial report

## Abstract

**Background/Objectives:** Clinical trials are essential in the development of new medical treatments, offering crucial data on their safety and effectiveness. **Methods:** This study provides a comprehensive analysis of clinical trials registered on ClinicalTrials.gov, examining the current landscape, challenges, and innovations that have shaped the field over the past century. Data were extracted on 7 March 2025 and analyzed to identify patterns in trial design, sponsorship, participant demographics, and geographical distribution. **Results:** The analysis reveals a continuous increase in clinical trial registrations, peaking in 2021, driven by the COVID-19 pandemic. Most trials focus on cancer, reflecting its global burden, with randomized controlled trials (RCTs) being the most common study design. However, challenges persist, including underrepresentation of certain demographics, limited global distribution, and insufficient reporting of trial results. Additionally, the underrepresentation of pediatric, elderly, and minority populations in trials limits the generalizability of findings. **Conclusions:** The analysis underscores the need for more inclusive and globally distributed research to address disparities in health outcomes.

## 1. Introduction

Clinical trials represent a critical step in the development of new medical treatments, providing essential data on their safety and effectiveness [1,2,3]. As the primary method through which new drugs, devices, and interventions are tested before they reach the general population, clinical trials play a pivotal role in advancing medical science and improving patient care [4]. The landscape of clinical trials encompasses numerous study designs, objectives, and methodologies, all tailored to answer specific scientific questions [5].

Over the years, the design and conduct of clinical trials have evolved significantly. Traditional randomized controlled trials (RCTs), long considered the gold standard, are now being complemented by adaptive designs, decentralized trials, and the use of real-world evidence [3]. These innovations are driven by a need to make trials more efficient, more patient-centered, and better suited to the complexities of modern medicine [6,7].

However, clinical trials face numerous challenges that can hinder their effectiveness and efficiency. Ethical considerations related to informed consent and the balance of risk and benefit are paramount. Additionally, recruiting and retaining participants remains a significant hurdle, particularly in trials involving minority populations, rare diseases, or pediatric and geriatric groups [8,9,10,11]. There is a need to understand the ever-changing landscape of clinical trials, which is driven by governmental regulatory policies, global epidemiology, and advances in medical research. This study provides a comprehensive overview of clinical trials, examining the current landscape, challenges, and innovations that are shaping the field today.

## 2. Materials and Methods

To assess the current landscape of clinical trials, we compiled a list of clinical trials and assembled corresponding information for each clinical trial using the ClinicalTrials.gov [12] database, a publicly accessible database managed by the National Institutes of Health (NIH). The dataset was extracted on 7 March 2025 and included all trials registered up to that date. Each clinical trial was identified by its unique National Clinical Trial (NCT) number, and data were collected including the NCT number, study status, study results, type of intervention, sponsor, collaborators, funder name, funder type, study design, trial phase, enrollment numbers, primary completion days, days to trial completion, available study documents, geographic distribution of trial, country, participants sex, age group and condition/disease for which clinical trial was conducted.

Once the data were downloaded from the ClinicalTrials.gov website, we added five new columns. First, we added a column “total duration of clinical trials” by subtracting the study start date from the study completion date for each clinical trial that was available in the data table extracted from the ClinicalTrials.gov webpage. We also added another new column, “days to primary completion”, using the study start date and primary completion date data. Additionally, wherever available, we also extracted country name information from the location column for each clinical trial and annotated each clinical trial with a new “country” column. For trials with multiple locations, we mined all the country names and annotated them with the NCT number. In addition, for single-country clinical trials, we also annotated each clinical trial to the corresponding “continent” in which it was conducted.

We used the search bar options “condition/disease” to extract clinical trials related to condition/disease that are listed on the 15 leading causes of death in the United States by the Centers for Disease Control and Prevention (CDC) [13]. The ranking of the disease list was based on the provisional mortality statistics as of 7 March 2025. We extracted the NCT number for those clinical trials that appeared on the search criteria under the “condition/disease” search bar for different diseases of a query. For the clinical trials that appeared in 2 or more criteria, we counted those clinical trials in the disease name they appeared. We annotated the disease name and added it to the master table. The condition/disease search terms and synonyms of conditions or diseases are listed in Supplementary Table S1.

Once the data were compiled, we selected interventional clinical trials from the start year 2005 onwards for our analysis. Data mining, analysis, and visualization were performed using different R packages using R version 3.6.3 [14] (R Foundation for Statistical Computing, Vienna, Austria) and R Studio version 2024.12.1+563.

The list of clinical trials and information associated with each trial is not exhaustive, as the list does not include other clinical trials that are not registered on the ClinicalTrials.gov webpage or trials that are registered on the webpage after 9 a.m. on 7 March 2025. Secondly, the list provided by ClinicalTrials.gov contains summaries that are abridged for public information and are not comprehensive in their entirety. Also, the list does not contain new updates to the existing clinical trials information after 7 March 2025.

## 3. Results

### 3.1. Number of Clinical Trials by Year

**Figure 1:**
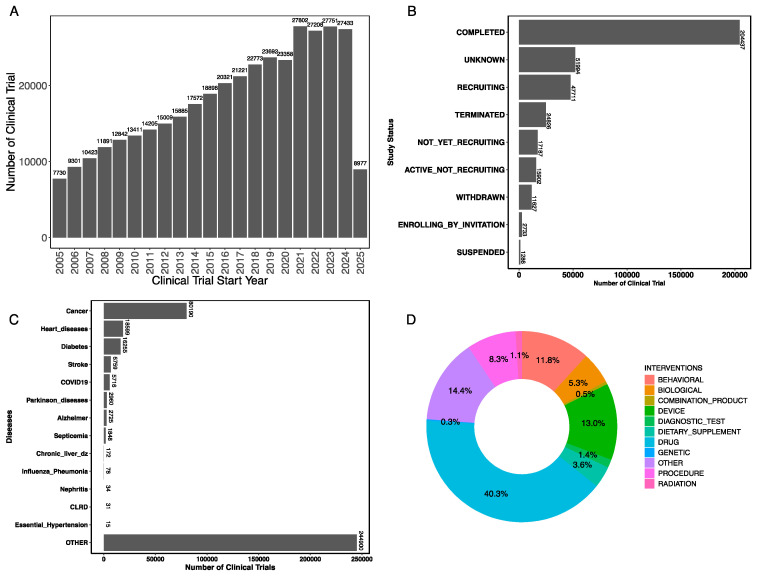
(**A**) Total number of clinical trials started in each year from 2005 to 2025 (current as of 7 March 2025); (**B**) study status of clinical trials; (**C**) number of clinical trials by disease/condition; (**D**) types of intervention/treatment.

Data from ClinicalTrials.gov showed an overall increase in the number of clinical trials spanning over the last two decades. On 7 March 2025, the total number of interventional clinical trials, hereafter referred to as clinical trials, available on the clinicaltrials.gov webpage was 404,637. The first recorded clinical trial dates back to 1900, and since then, the number of registered trials has grown steadily, with a significant peak in 2021 during the COVID-19 pandemic, during which 27,802 trials were initiated, followed by 27,751 clinical trials initiated in 2023 (Figure 1A). In this figure, we plot a number of clinical trials started in each year from 2005 to 2025. Other clinical trials with missing start dates and clinical trials with start dates beyond 2025 were not shown in Figure 1A.

### 3.2. Study Status of Clinical Trials

Among the trials analyzed, the majority (204,437) have been completed, while others are in various stages, including recruiting (47,711), terminated (24,826), not yet recruiting (17,187), and enrolling by invitation (2733) (Figure 1B). This distribution reflects the dynamic nature of clinical research, where trials are continuously starting, progressing, concluding, or terminating.

### 3.3. Clinical Trials by Disease/Condition

We annotated each clinical trials to 15 leading causes of death in the United States, as reported by the Centers for Disease Control and Prevention (CDC), we found cancer-related trials represent the largest category of studies by disease (80,190), reflecting the high burden of this disease and the ongoing efforts to develop effective treatments. Other significant categories include clinical trials focused on heart disease (18,599), diabetes (16,255), and stroke (6759). A substantial number of trials address conditions that are not among the leading causes of death, grouped as OTHER, highlighting the broad scope of clinical research (Figure 1C).

### 3.4. Types of Intervention/Treatment

Drug-related interventions are the focus of major clinical trials, accounting for 40.3% of all trials. Other types of interventions include device (13%), behavioral (11.8%), procedural interventions (8.3%), biological (5.3%), diagnostic test (1.4%), dietary supplements (3.6%), and radiation (1.1%), as shown in Figure 1D. The diversity of intervention types illustrates the multifaceted nature of clinical research, which spans pharmacological, behavioral, and technological approaches to treatment.

### 3.5. Purpose of Interventional Trials

**Figure 2:**
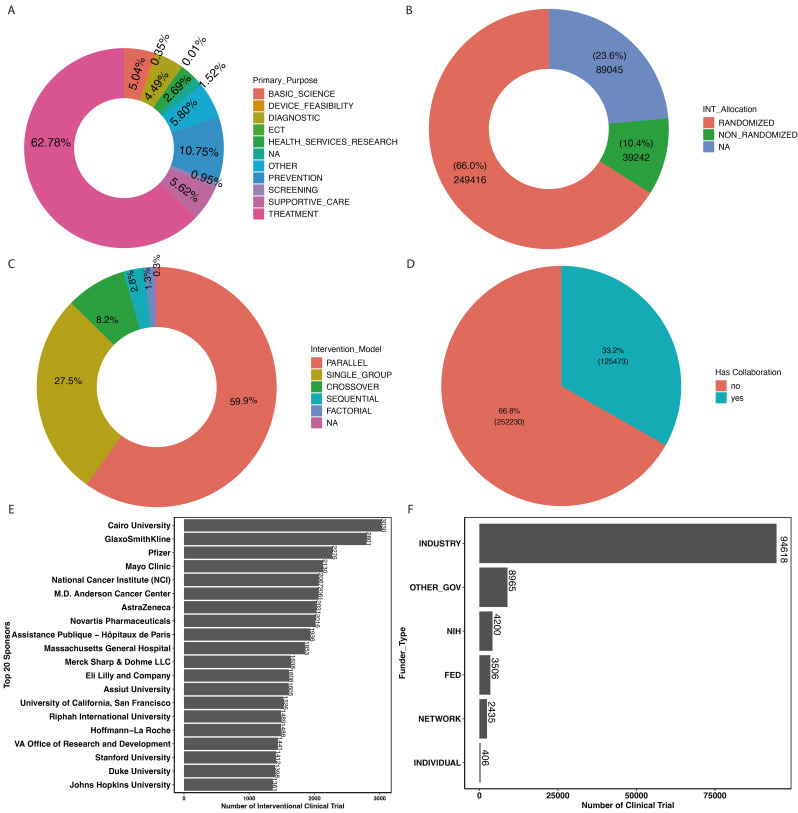
(**A**) Distribution of clinical trials by their primary purpose; (**B**) interventional clinical trials by allocation; (**C**) distribution of interventional study model; (**D**) distribution of clinical trials by collaboration status; (**E**) top 20 sponsors of interventional clinical trials; (**F**) distribution of funder type for the clinical trials conducted in last two decades.

For interventional type clinical trials, the major primary purpose is treatment (62.78%), followed by prevention (10.75%), supportive care (5.61%), basic science (5.04%), diagnostic (4.49%), health services research (2.691%), screening (0.95%), device feasibility (0.35%), and ECT (0.01%) as shown in Figure 2A. These proportions highlight the central role of clinical trials in developing new treatments and preventive measures for various health conditions.

### 3.6. Randomization and Interventional Model

For interventional type clinical trials, randomized controlled trials (RCTs) remain the dominant study design, accounting for 66% of the trials, whereas non-randomized trials accounted for 10.4% (Figure 2B). Also, the parallel model, being the most common study model, accounted for 59.9% of the clinical trials, followed by the single group model (27.4%) and the crossover model (8.2%), as shown in Figure 2C.

### 3.7. Collaborations, Sponsorship, and Funder Type

Approximately 33.2% of all clinical trials involve collaborations between multiple institutions or organizations (Figure 2D). Industry sponsors, particularly pharmaceutical companies, play a leading role in funding and conducting clinical trials, with GlaxoSmithKline being the most prominent sponsor. Government and academic institutions such as the Mayo Clinic, National Cancer Institute (NCI), and MD Anderson Cancer Center contribute significantly to clinical trial sponsorship. The top 20 sponsors for clinical trials are summarized in Figure 2E. Overall, industry sponsorship is the predominant source of funding for clinical trials, reflecting the commercial interest in developing new therapies. Various governmental institutions, including the National Institutes of Health (NIH), are major non-industry sponsors, particularly for trials focused on public health priorities (Figure 2F).

### 3.8. Trial Phases and Enrollments

**Figure 3:**
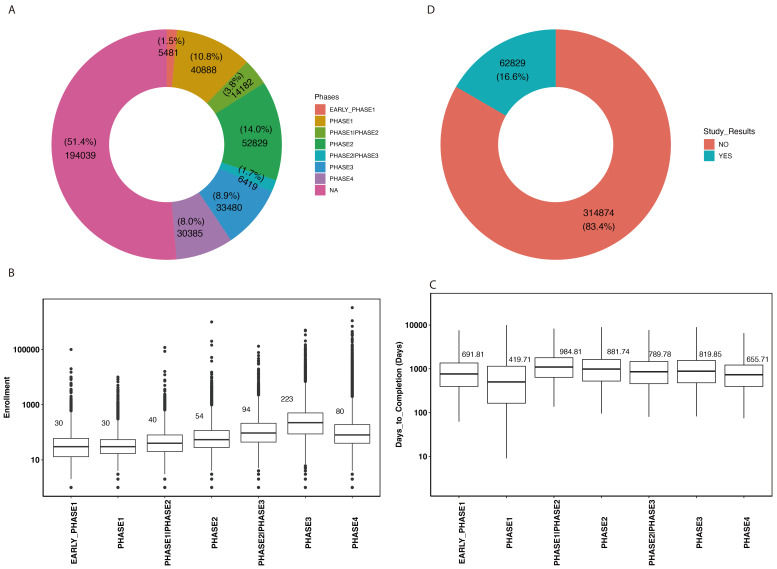
(**A**) Overall distribution of clinical trials phases; (**B**) distribution of enrollment number by trial phase; (**C**) distribution of days to completion; (**D**) clinical trials with and without study results.

For the clinical trials with phase information, excluding the clinical trials where phase is not available, the majority of clinical trials are on phase 2, followed by phase 1, phase 3, phase 4, and other phases (Figure 3A). Phase 3 trials, which are critical for determining the efficacy of a treatment before it is approved for general use, have the largest median number of participants (Figure 3B). This phase is crucial for ensuring that new treatments are safe and effective for a broad population.

### 3.9. Clinical Trial Duration, Reporting, and Documentation

We observed that the median time to primary completion varies by trial phase, with phase 1 generally being shorter, with 426 days (Supplementary Figure S1). Similarly, median time to completion varies by trial phase, with phase 1 generally being shorter, with 419 days (Figure 3C). Notably, only 16.6% of clinical trials have reported their results on ClinicalTrials.gov (Figure 3D). This indicates a gap in reporting and the dissemination of research findings to the ClinicalTrials.gov database.

### 3.10. Participant Demographics and Geographical Distribution

**Figure 4:**
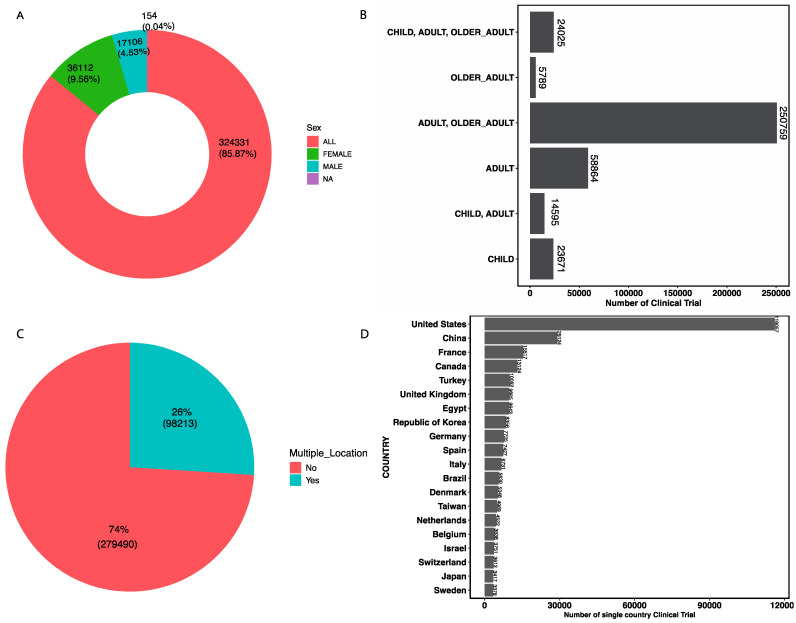
(**A**) Overall distribution of clinical trial participants by sex; (**B**) overall distribution of clinical trial participants by age group; (**C**) clinical trials with multiple locations; (**D**) distribution of clinical trials conducted in a single country.

**Figure 5:**
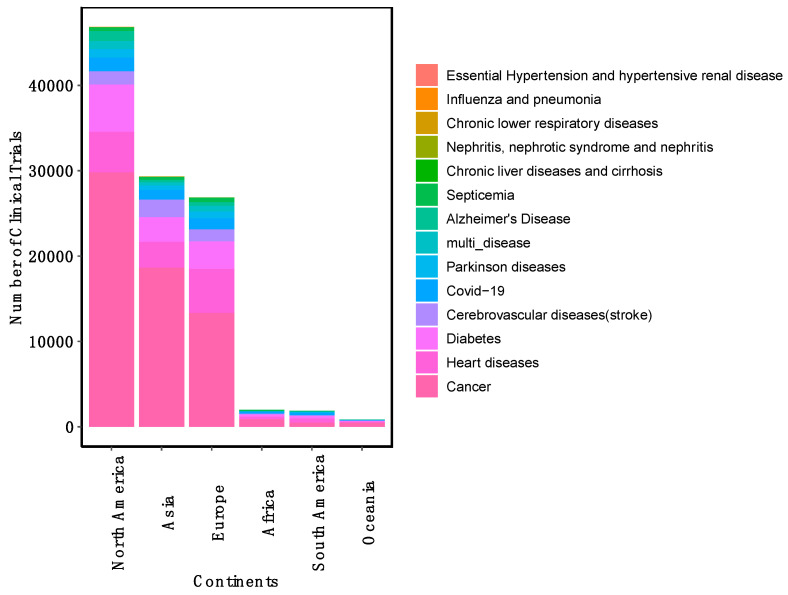
Distribution of clinical trials by continents and disease for the last two decades.

A total of 85.87% of clinical trials include both male and female participants, 9.56% of trials were exclusively for female participants, and 4.53% of trials were exclusively for male participants (Figure 4A). In relation to the age group of the clinical trial participants, there is a significant focus on the adult and older adult age group, followed by adult adult-only age group (Figure 4B). The majority of clinical trials were conducted in a limited geographical distribution. Only 26% of clinical trials were conducted in multiple locations (Figure 4C), and only 8.2% of the total clinical trials were multi-national clinical trials (Supplementary Figure S2), which suggests barriers to international collaboration in clinical research. Notably, the United States hosts the majority of clinical trials, followed by China, France, Canada, Turkey, and the United Kingdom (Figure 4D). For the top 15 highest mortality-causing diseases, we annotated the single-country trials to their respective continents in which clinical trials were carried out. Overall, we found clinical trials for these diseases were predominantly in North America, followed by Asia and then Europe (Figure 5).

We were not able to access the race and ethnicity distribution of participants. However, there is a noticeable underrepresentation of pediatric and elderly participants, as well as minority populations and low-income countries.

### 3.11. Landscape of Clinical Trials in the Last Two Decades

**Figure 6:**
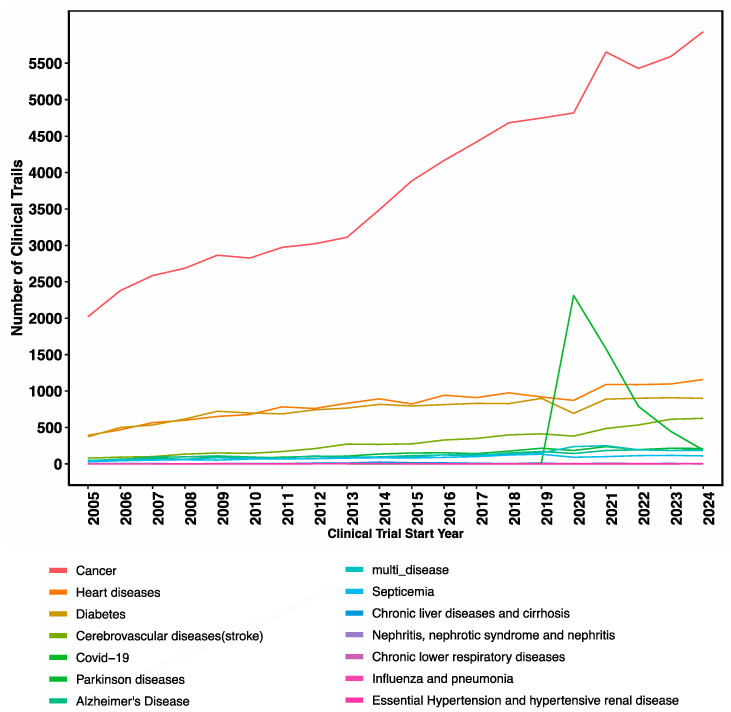
Landscape of clinical trials in the last two decades by disease/condition.

Data analysis of clinical trials with start dates from 2005 to 2024 annotated to disease or conditions revealed a consistent focus on cancer-related trials in the last two decades, reflecting ongoing efforts to develop new treatments for this complex disease. Additionally, there was a notable surge in the COVID-19 trials during the last COVID-19 pandemic, underscoring the impact of global health emergencies on clinical research priorities (Figure 6).

## 4. Discussion

This comprehensive analysis of clinical trials registered on ClinicalTrials.gov provides valuable insights into the current landscape of clinical trials, challenges, and opportunities in clinical research over the past century. The steady increase in the number of trials reflects the growing complexity of modern medicine and the need for rigorous evaluation of new therapies. The peak in trial registrations in 2021 is particularly noteworthy and can be attributed to the global response to the COVID-19 pandemic, which catalyzed a surge in research activity [15].

The analysis of trial statuses highlights the dynamic nature of clinical research. While a significant number of trials have been completed, a substantial proportion remains ongoing, with some being terminated or suspended. These findings underscore the challenges inherent in clinical trial conduct, including issues related to participant recruitment, funding, and regulatory approval [16]. The predominance of cancer-related trials aligns with the high global burden of this disease, but it also raises concerns about the equitable distribution of research resources. Diseases that are less common or less commercially viable may not receive the same level of research attention, potentially leaving significant gaps in medical knowledge and treatment options [17,18]. The focus on drug-related interventions and the prevalence of randomized controlled trials reflects the central role of pharmaceuticals and rigorous testing methodologies in clinical research. However, the growing interest in other types of interventions, such as behavioral and procedural, indicates a broadening of research focus to include non-pharmacological approaches to health and disease management.

The analysis of funding and sponsorship reveals the dominance of industry sponsors, which suggests a strong commercial influence on clinical research. While industry sponsorship is crucial for advancing drug development and bringing new therapies to market, it also raises concerns about potential conflicts of interest and the focus of research on commercially viable products rather than public health needs [19]. Previous studies have also reported that studies on clinical trials are prominently industry-sponsored, and conclusions from these studies favor sponsors [20]. Government and academic institutions play a significant role, particularly in funding research that addresses less profitable but equally important health issues, such as rare diseases and public health interventions.

One of the major challenges identified in the analysis is the underrepresentation of certain demographic groups, particularly pediatric, elderly, and a lack of data reporting on race and ethnicity. This lack of diversity in clinical trial participants limits the generalizability of research findings and can lead to disparities in treatment outcomes across different population groups. Ensuring that clinical trials are inclusive and representative is critical for developing therapies that are effective and safe for all segments of the population [21]. The geographical distribution of clinical trials shows a concentration in high-income countries [22,23], particularly the United States. While this reflects the strong research infrastructure and funding available in these regions, it also highlights the barriers to conducting clinical research in low- and middle-income countries. These barriers include limited resources, regulatory challenges, and difficulties in recruiting and retaining participants. Expanding clinical research to these regions is essential for addressing global health challenges and ensuring that treatments are relevant and accessible to diverse populations worldwide [24]. The total number of clinical trials observed since 2005, particularly the increase in cancer and COVID-19-related trials, reflects the responsiveness of the clinical research community to emerging health challenges. The surge in trials during the COVID-19 pandemic is a clear example of how global health emergencies can drive research priorities and lead to rapid advancements in the understanding and treatment of infectious diseases [15].

Despite these advances, there remains a significant gap in the reporting of trial results. A previous study has also reported a lack of reporting results to the database and highlighted the need for an up-to-date database for the dissemination of clinical trial results [25].

The finding that only 16.6% of trials have reported their results on ClinicalTrials.gov highlights a critical issue in the reporting and dissemination of clinical research in the central database. Improving the reporting and sharing of trial results is essential for maximizing the impact of clinical research [26] and ensuring that findings contribute to the broader body of medical knowledge.

The future of clinical trials will likely be shaped by innovations aimed at addressing the current challenges in trial design, trial method, sample size, and patient demographics to make the clinical trials more reliable and evidence-based on real-world data [27]. Some of the most promising directions for future research and development in clinical trials will be towards personalized medicine and precision medicine, decentralized and virtual trials, integration of real-world evidence (RWE), and adaptive trial designs [28], global collaboration and harmonization, ethical and regulatory innovations, and enhanced participant engagement [29].

## 5. Conclusions

In conclusion, the landscape of clinical trials is rapidly evolving, driven by advances in technology, a greater emphasis on patient-centered care, and the need to address complex and emerging health challenges. Addressing regulatory and scientific concerns, such as patient enrollment, trial design, sample size, operational and ethical challenges, clinical trials will continue to advance, ultimately leading to more effective, safe, and equitable treatments for patients worldwide.

## Data Availability

The most up-to-date version of the clinical trials data is publicly available at https://clinicaltrials.gov/ (accessed on 7 March 2025).

## Supplementary Materials

The following supporting information can be downloaded at: https://www.mdpi.com/article/10.3390/jcm14072519/s1, Table S1: Synonyms of conditions or diseases. Figure S1: Distribution of days to primary completion (in days) by trial phase; Figure S2: Distribution of multinational clinical trials.
