# Supplementary material for: The Current Landscape of Clinical Trials
Source: J Clin Med. 2025 Apr 7;14(7):2519. doi: 10.3390/jcm14072519 (PMC11989625; doi:10.3390/jcm14072519)
Supplement: Supplementary file 1 [file jcm-14-02519-s001.zip › TableS1.pdf]

Table S1: Synonyms of conditions or diseases

SN	Disease/Condition	synonyms of conditions or diseases
1	Heart diseases	heart; Cardiac; coronary; Hearts; Cardiac structure; Heart structure<br><br>heart diseases; Heart disease; Cardiac Disease; Cardiac Diseases; cardiopathy; Cardiac disorder; disease cardiac; disorder heart; cardiac disorders; cardiopathies; heart disorder; syndrome heart disease; diseases of the heart<br><br>diseases; Disease; Disorders; disorder; Diagnosis; condition; disease type; Other Disease
2	Cancer	cancer; Neoplasms; Tumors; Tumor; Neoplasm; malignancies; Cancers; Oncology; Malignancy; Tumour; tumours; Neoplasia; Malignant neoplasm; malignant tumors; Malignant tumor; Malignant Neoplasms; primary cancer; Neoplastic Disease; Cancer NOS; neoplasias; neoplastic syndrome; Primary Malignant Neoplasm; tumor NOS; Malignant tumour; Malignant neoplastic disease; malignant tumours; malignant neoplasm primary
3	Cerebrovascular diseases (stroke)	stroke; Cerebrovascular accident; cerebral stroke; Strokes; cerebrovascular stroke; cerebral vascular accident; Apoplexy; Brain attack; Cerebrovascular Accidents; Stroke Syndrome; Cerebrovascular Apoplexy; stroke cerebral; Stroke/cerebrovascular accident; Brain Vascular Accident; Cerebral vascular events; Stroke NOS
4	Chronic lower respiratory diseases	lower; lowering<br><br>respiratory; respiratory tract<br><br>respiratory diseases; Respiration Disorders; respiratory disease; respiratory disorder<br><br>diseases; Disease; Disorders; disorder; Diagnosis
5	Alzheimer diseases	alzheimer disease; Alzheimer's Disease; Alzheimers Disease; Alzheimer Dementia; Alzheimer's Dementia; dementia alzheimers; Dementia of the Alzheimer's type;

		Senile Dementia; Alzheimer Type Dementia; Familial Alzheimer disease; Dementia Alzheimer's type; Alzheimer Type Senile Dementia; alzheimer's diseases; familial alzheimer's disease; pN2; dats; Alzheimer Syndrome<br><br>disease; Disorders; Diseases; disorder; Diagnosis; disease type; condition
6	Diabetes	diabetes; Diabetes Mellitus; diabete mellitus; diabetes NOS
7	Nephritis, nephrotic syndrome and nephritis	nephritis; Inflammation of kidney; kidney inflammation<br><br>nephrotic syndrome; Nephrosis; Nephrotic Syndromes<br><br>syndrome; Syndromes
8	Chronic liver diseases and cirrhosis	chronic; Chronic graft versus host disease; Chronic GVHD<br><br>liver; Livers<br><br>liver diseases; Liver Disease; hepatic disease; Hepatopathy; Hepatic Diseases; liver disorder; Disorder of liver<br><br>diseases; Disease; Disorders; Diagnosis; disorder; condition; disease type<br><br>cirrhosis; Liver Cirrhosis; Cirrhosis liver; Hepatic Cirrhosis; cirrhosis of the liver; Cirrhosis of liver<br><br>cirrhosis liver; Cirrhosis; Liver Cirrhosis; Hepatic Cirrhosis; cirrhosis of the liver; Cirrhosis of liver
9	Covid-19	covid; SARS-CoV-2; Severe Acute Respiratory Syndrome Coronavirus 2; Novel Coronavirus; SARS-CoV-2 Virus; COVID19 Virus; 2019-nCoV; 2019 novel coronavirus; SARS Coronavirus 2; SARS2; COVID 19 Virus; SARS CoV 2 Virus; Wuhan Coronavirus

10	Influenza and pneumonia	influenza; Influenza Vaccine; Flu; Influenza vaccines; Influenza virus vaccine; Flu vaccine; Fluzone; trivalent influenza vaccine; Fluarix; flumist; Human Influenza; Flu vaccination; influenza virus vaccine trivalent; Fluviral; Flu shot; Grippe; Influenza in Humans; Influenza Virus Vaccines; flu vaccines; Fluzone HD; FluLaval; fluvirin; Human Flu; Flucelvax; flus; Influenza in Human; flu syndrome; Trivalent Live-Attenuated Influenza Vaccine; Agriflu; Influenza NOS; flushield<br><br>pneumonia; Pneumonias; inflammation lungs
11	Essential Hypertension and hypertensive renal disease (essential hypertension OR hypertension renal disease)	essential hypertension; Primary hypertension; hypertension primary<br><br>hypertension; High blood pressure; arterial hypertension; Blood pressure high; Hypertension arterial; Elevated blood pressure; vascular hypertension; HTN - Hypertension; Hypertensive disease; hypertensive disorder; systemic hypertension; hyperpiesia; vascular hypertensive disorder; HBP; High Blood Pressures; hyperpiesis<br><br>hypertension renal disease; hypertensive kidney disease; hypertensive nephropathy; Hypertensive renal disease; Hypertension secondary to renal disease<br><br>renal; Kidney; Kidneys; Ren<br><br>renal disease; Kidney Diseases; Kidney disease; Nephropathy; nephropathies; kidney disorder; renal diseases; Renal disorder<br><br>disease; Diseases; Disorders; disorder; Diagnosis; condition; Other Disease
12	Septicemia	septicemia; Sepsis; toxemia; Bloodstream Infection; Bloodstream Infections; Sepsis Syndrome; Septicaemia; systemic infection; Systemic infections; Infection systemic; Systemic Sepsis
13	Parkinson diseases	parkinson's; Idiopathic Parkinson's disease; parkinson s disease; idiopathic parkinson disease; Primary Parkinsonism; parkinson's syndrome; Lewy Body Parkinson's Disease; Lewy Body Parkinson Disease; Paralysis Agitans; Shaking palsy; parkinson disease lewy body<br><br>diseases; Disease; Disorders; disorder; Diagnosis; condition
