# Supplementary figures and images for: The Current Landscape of Clinical Trials
Source: J Clin Med. 2025 Apr 7;14(7):2519. doi: 10.3390/jcm14072519 (PMC11989625; doi:10.3390/jcm14072519)

Days\_to\_Primary\_Completion (Days)

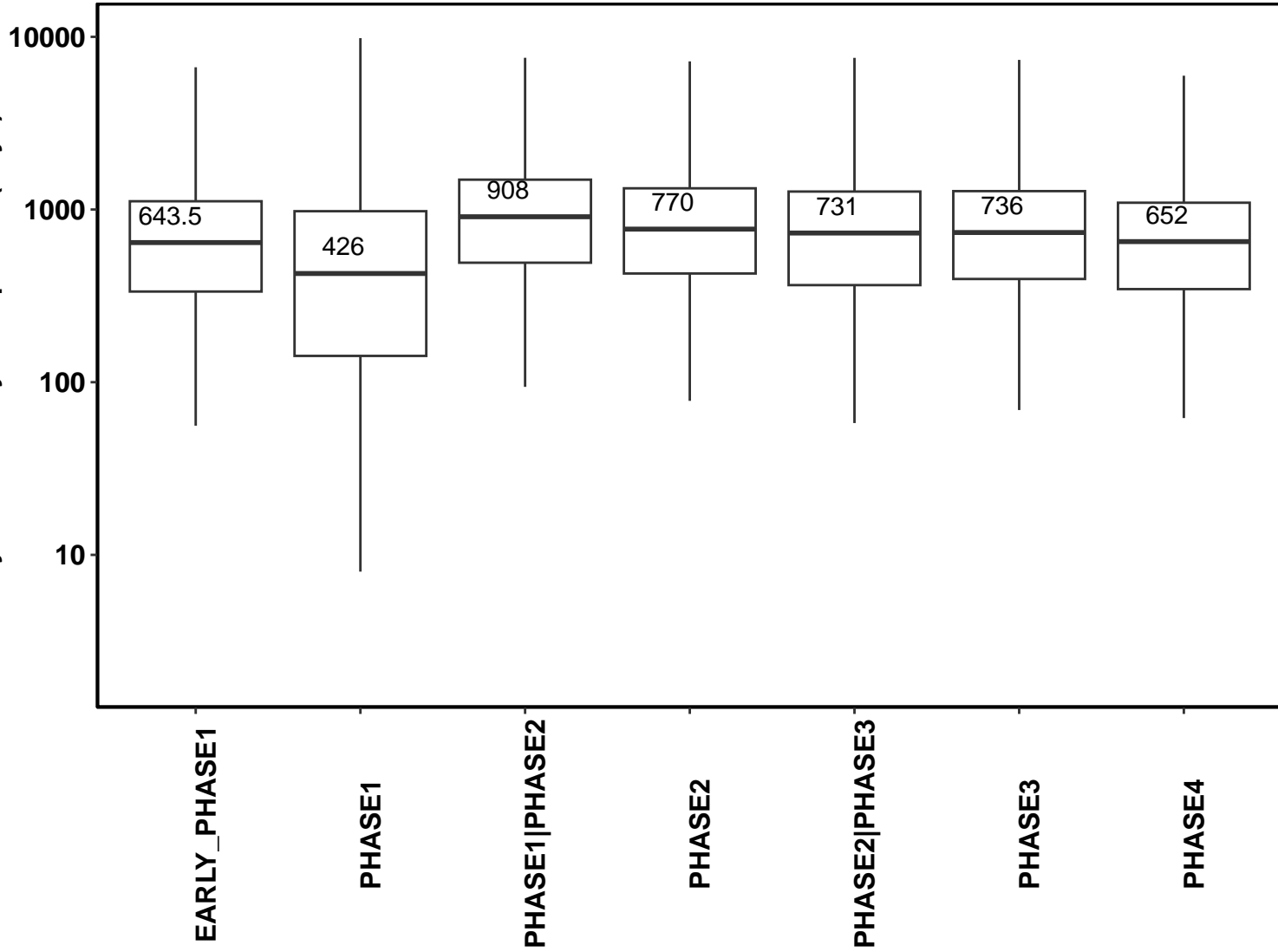

Supplement: Supplementary file 1 [file jcm-14-02519-s001.zip › SF1.pdf]

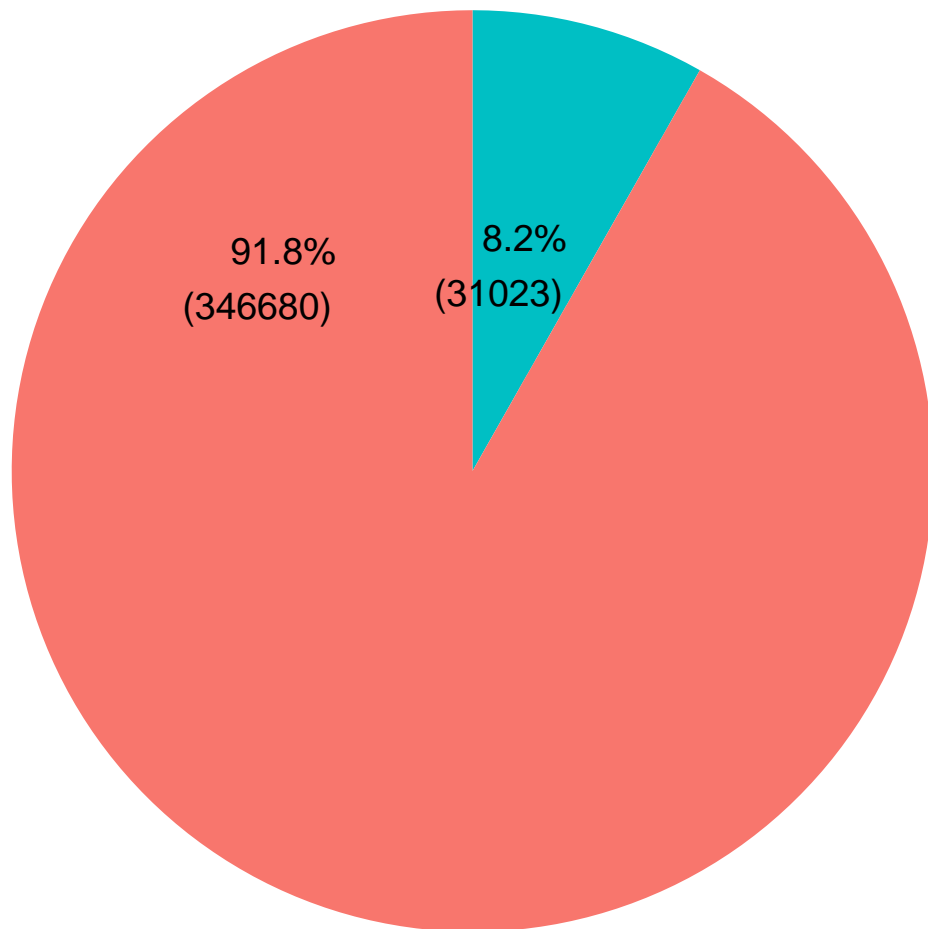

Multi\_National\_Trial

No

Yes

Supplement: Supplementary file 1 [file jcm-14-02519-s001.zip › SF2.pdf]
